# Molecular characterization of the HIV-1 *gag *nucleocapsid gene associated with vertical transmission

**DOI:** 10.1186/1742-4690-3-21

**Published:** 2006-04-06

**Authors:** Brian P Wellensiek, Vasudha Sundaravaradan, Rajesh Ramakrishnan, Nafees Ahmad

**Affiliations:** 1 Department of Microbiology and Immunology, College of Medicine, The University of Arizona Health Sciences Center, Tucson, Arizona, USA

## Abstract

**Background:**

The human immunodeficiency virus type 1 (HIV-1) nucleocapsid (NC) plays a pivotal role in the viral lifecycle: including encapsulating the viral genome, aiding in strand transfer during reverse transcription, and packaging two copies of the viral genome into progeny virions. Another *gag *gene product, p6, plays an integral role in successful viral budding from the plasma membrane and inclusion of the accessory protein Vpr within newly budding virions. In this study, we have characterized the *gag *NC and p6 genes from six mother-infant pairs following vertical transmission by performing phylogenetic analysis and by analyzing the degree of genetic diversity, evolutionary dynamics, and conservation of functional domains.

**Results:**

Phylogenetic analysis of 168 *gag *NC and p6 genes sequences revealed six separate subtrees that corresponded to each mother-infant pair, suggesting that epidemiologically linked individuals were closer to each other than epidemiologically unlinked individuals. A high frequency (92.8%) of intact open reading frames of NC and p6 with patient and pair specific sequence motifs were conserved in mother-infant pairs' sequences. Nucleotide and amino acid distances showed a lower degree of viral heterogeneity, and a low degree of estimates of genetic diversity was also found in NC and p6 sequences. The NC and p6 sequences from both mothers and infants were found to be under positive selection pressure. The two important functional motifs within NC, the zinc-finger motifs, were highly conserved in most of the sequences, as were the *gag *p6 Vpr binding, AIP1 and late binding domains. Several CTL recognition epitopes identified within the NC and p6 genes were found to be mostly conserved in 6 mother-infant pairs' sequences.

**Conclusion:**

These data suggest that the *gag *NC and p6 open reading frames and functional domains were conserved in mother-infant pairs' sequences following vertical transmission, which confirms the critical role of these gene products in the viral lifecycle.

## Background

Mother-to-infant (vertical) transmission of HIV-1 occurs at a rate of 30%, and accounts for 90% of infections in children worldwide. Transmission of the virus can occur at three stages: prepartum (in utero), intrapartum (during birth), and postpartum (breast feeding). Several factors have been linked to vertical transmission including: low CD4 count and high viral load of the mother, advanced maternal disease status, invasive procedures, infections during pregnancy and prolonged exposure of the infant to blood and ruptured membranes during birth [1-8]. The exact molecular mechanisms of vertical transmission are not well understood, however we and others have shown that the minor HIV-1 genotypes are transmitted from mother to infant [9,10]. It has also been shown that the macrophage-tropic (R5) phenotype is involved in transmission [11]. Analysis of several HIV-1 accessory and regulatory genes, including *vif, vpr, vpu, nef, tat and rev *has revealed conservation of functional domains of these genes during vertical transmission [12-17]. In addition, transmitting mothers' *vif and vpr *sequences were more heterogeneous and the functional domain more conserved than non-transmitting mothers' sequences [12-17]. However, other HIV-1 genes may also play a crucial role in virus transmission and pathogenesis.

One such gene product, the *gag *nucleocapsid (NC) plays a pivotal role in the viral lifecycle, including encapsulating the viral genome, aiding in the reverse transcription process, protecting the viral genome from nuclease digestion and packaging two copies of the viral genome into progeny virions [18-23]. The NC gene product, also termed p7, is translated as a Pr55 Gag precursor and when cleaved is 55 amino acids long. It contains one major functional domain, consisting of two zinc finger like motifs. These motifs allow the NC to bind the packaging signal, or Ψ site, on viral RNA, as well as coat the viral genome [18,24,25]. They contain the sequence C-X_2_-C-X_4_-H-X_4_-C with the critical residues consisting of three cystines and one histidine [20]. When these critical zinc binding amino acids are mutated to non-zinc binding residues, it results in virions that are defective in RNA packaging and replication [18,21,26]. Several basic amino acid residues throughout the NC gene product are also associated with RNA binding, and aid in NC function [18,21]. These basic residues are responsible for interaction with the side chains of the viral nucleic acids. NC plays several roles during the reverse transcription step of the HIV-1 lifecycle. It is responsible for ensuring proper annealing of the tRNA^Lys ^primer to the primer binding site to initiate reverse transcription, and also aids in strand transfer so that reverse transcription can continue [20,21,23,27,28]. During and after reverse transcription, it has been shown that NC binds to the newly generated viral DNA and protects it from cellular nucleases until it can integrate into the host cell genome [22,29]. Due to the importance of this gene any alterations to the NC may affect transmission and pathogenesis of the virus.

Another example of a crucial gene product is p6, which plays an integral role in successful viral budding from the plasma membrane and inclusion of the accessory protein Vpr within newly budding virions [30-35]. The p6 gene product is also initially translated as a Pr55 Gag precursor and is 52aa long when cleaved by the viral protease. The p6 protein contains a viral late (L) domain with the sequence PTAPP, which is necessary for viral budding [36,37]. It has been shown that the late domain interacts with the host cell factor Tsg101 which is involved in regulating intracellular trafficking [32,35,38,39]. The late domain has also been shown to be crucial for detachment of virions from the host cell surface. Defects and mutations in the late domain can result in chains of immature virions that cannot release from the host cell surface [36,40]. The p6 gene product also contains a region with the sequence DKELYPLASLRSLFG that is responsible for interacting with the host cell factor AIP1 [31,41,42]. AIP1 has been shown to interact with Tsg101 and host factor ESCRT-III to function in a late-acting endosomal sorting complex that is essential for viral budding [31,41-43]. There are two domains that could possibly be required for inclusion of Vpr, either the FRFG domain [30] or the (LXX)_4 _domain [33,34,44,45]. Defects within the Vpr binding domains could result in virions that lack Vpr. This would affect the ability of the virus, upon infection, to replicate in nondividing cells such as macrophages, and would affect the ability of the viral DNA to localize to the host cell nucleus for integration. The p6 gene product is also critical in the viral lifecycle, and therefore any changes within it may effect the transmission and pathogenesis of the virus.

In this study, we have characterized and analyzed the genetic diversity and population dynamics of the *gag *NC and p6 genes from six mother-infant pairs following vertical transmission. Our findings suggest that these gene products are mostly conserved during mother-infant transmission. Furthermore, the critical functional domains were conserved in most sequences analyzed. These results help to further our understanding of the molecular mechanisms that are involved in vertical transmission of HIV-1.

## Results

### Phylogenetic analysis of NC and p6 sequences from mother-infant pairs

**Figure 1:**
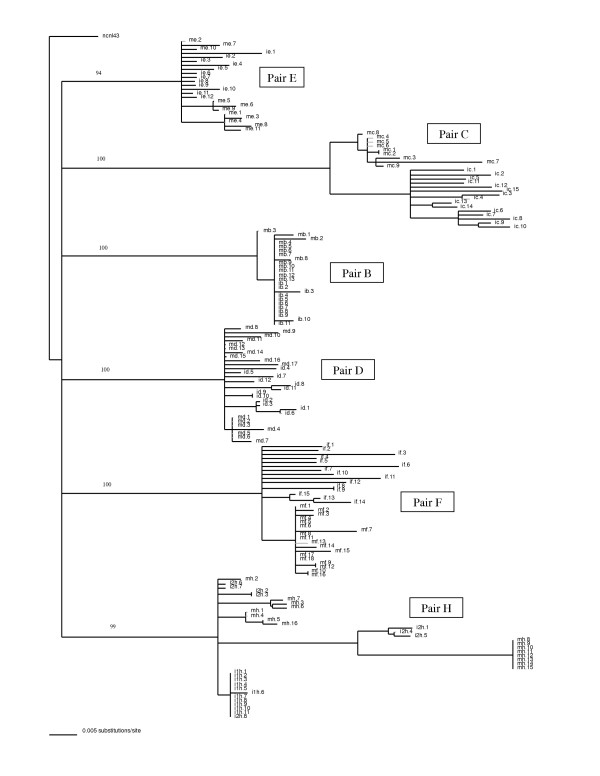
Phylogenetic analysis of 168 HIV-1 NC and p6 sequences from six mother-infant pairs; pairs B, C, D, E, F, and H. The neighbor-joining tree is based on the distance calculated between the nucleotide sequences from the six mother-infant pairs. Each terminal node represents one sequence. The values on the branches represent the occurrence of that branch over 1,000 bootstrap resamplings. Each pair formed a distinct subtree, and within each subtree the mother and infant sequences were generally separated into clusters, although some intermingling was observed. The formation of subtrees indicated that epidemiologically linked mother-infant pairs were closer to each other evolutionarily than to epidemiologically unlinked pairs, and that there was no PCR cross-contamination. The placement of the HIV-1 lab control strain NL4-3 indicates that no PCR contamination occurred.

Multiple independent polymerase chain reactions (PCRs) were performed on peripheral blood mononuclear cell (PBMC) DNA from six mother-infant pairs, a total of 13 patients including one mother who gave birth to HIV-1 positive twins. Eight to eighteen clones from each patient were obtained and sequenced. The phylogenetic analysis was performed using a neighbor-joining tree of the 168 NC and p6 sequences from the mother-infant pairs (Fig. 1). This neighbor-joining tree was generated by incorporating a best-fit model of evolution into PAUP [46], and the resulting tree was then bootstrapped 1000 times to ensure fidelity. Analysis of the tree demonstrated that the sequences from the six mother-infant pairs form distinct, well separated subtrees, and all pairs were separate from the lab control strain HIV-1 isolate NL4-3. Within each subtree the sequences for the mother and infant are generally well separated into subtrees, however some intermingling was observed in pairs B, D, E, and H. The intermingling of mother-infant sequences suggests that the isolates from these patients are very closely related, and had not as of yet evolved to form separate, distinct subtrees. Taken together the data indicates that epidemiologically linked (mother-infant) patient sequences are closer to each other evolutionarily than epidemiologically unlinked sequences. The separation of the mother-infant sequences from each pair and NL4-3 indicates that no PCR contamination occurred.

### Coding potential of NC and p6 gene sequences

**Figure 2:**
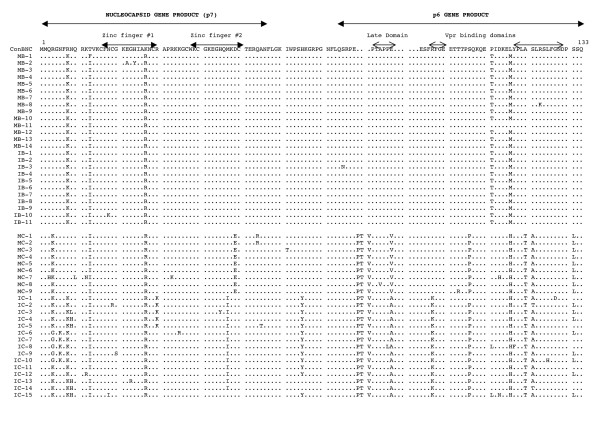
Multiple sequence alignment of deduced amino acids of NC and p6 from mother-infant pairs B and C. Within the alignment, the top sequence is the NC consensus B (ConBNC) sequence to which the mother-infant pair sequences are compared. Each line of the alignment represents one clone sequence, and is identified by a clone number with M referring to mother and I referring to infants. The dots represent agreement with the consensus sequence, while substitutions are represented by a single letter amino acid code. Stop codons are shown as asterisks (*). The functional domains within the sequence are indicated above the alignment.

**Figure 3:**
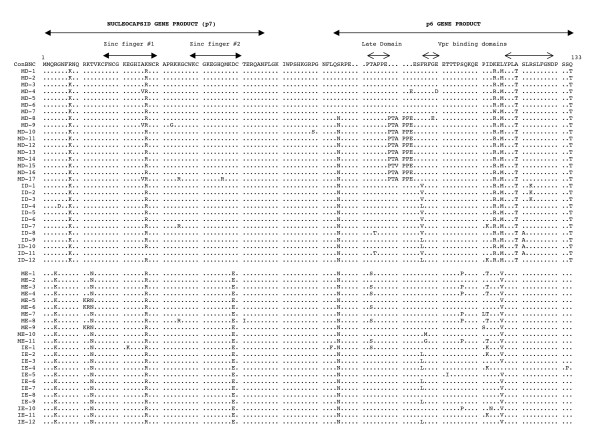
Multiple sequence alignment of deduced amino acids of NC and p6 from mother-infant pairs D and E. Within the alignment, the top sequence is the NC consensus B (ConBNC) sequence to which the mother-infant pair sequences are compared. Each line of the alignment represents one clone sequence, and is identified by a clone number with M referring to mother and I referring to infants. The dots represent agreement with the consensus sequence, while substitutions are represented by a single letter amino acid code. Stop codons are shown as asterisks (*). The functional domains within the sequence are indicated above the alignment.

**Figure 4:**
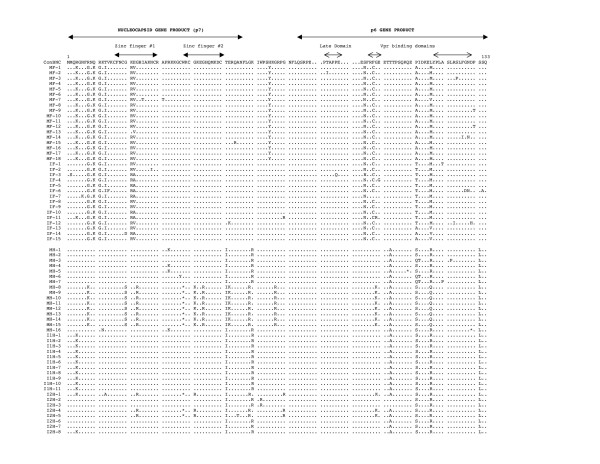
Multiple sequence alignment of deduced amino acids of NC and p6 from mother-infant pairs F and H, including both infant H twins (I1H and I2H). Within the alignment, the top sequence is the NC consensus B (ConBNC) sequence to which the mother-infant pair sequences are compared. Each line of the alignment represents one clone sequence, and is identified by a clone number with M referring to mother and I referring to infants. The dots represent agreement with the consensus sequence, while substitutions are represented by a single letter amino acid code. Stop codons are shown as asterisks (*). The functional domains within the sequence are indicated above the alignment.

The multiple sequence alignment of the deduced amino acid sequences of the HIV-1 NC and p6 genes is shown in Figs. 2, 3, 4. Of the 168 sequences analyzed, 156 contained an intact open reading frame (ORF), yielding a frequency of 92.8%. This high frequency indicates that the coding potential of the NC and p6 genes was maintained in most of the sequences analyzed. Looking more closely, the frequency of an intact ORF for the mothers' sequences was 89.4%, while the infants' sequences yielded a frequency of 96.3%. Several clones within mother-infant pair H were found to be defective due to a single nucleotide substitution, insertion or deletion, which resulted in the formation of a stop codon. There were several patient and pair specific sequence patterns within the NC sequences analyzed. An insertion of proline-threonine-valine (PTV) was seen in the sequences of mother-infant pair C at position 78, and an insertion of proline-threonine-alanine-proline-proline-glutamate (PTAPPE) was observed within several sequences of mother D at position 84. This resulted in a duplication of the PTAP motif within this patient. An amino acid substitution was also present in most of the sequences when compared as a whole, a leucine (L) was replaced with a methionine (M), valine (V), histidine (H), arginine (R) or glutamine (Q) at position 116.

### Variability of NC and p6 gene sequences in mother-infant pairs

The nucleotide and amino acid distances, which measure the degree of genetic variability based on pairwise comparison, were calculated for the six mother-infant pairs' sequences (Table 2). The nucleotide sequences within mothers B, C, D, E, F, and H varied by 0.26, 0.53, 0.84, 1.13, 0.27, and 5.04% (median values) respectively, ranging from 0 to 6.30%. The infant (B, C, D, E, F, I1H, and I2H) sequences differed by 0, 2.59, 0.88, 1.11, 1.78, 0, 3.22% (median values) respectively, ranging from 0 to 5.03%. Moreover, the nucleotide sequence variability between epidemiologically linked mother-infant pairs (pairs B, C, D, E, F, and H) varied by 0, 3.16, 1.13, 1.12, 1.99, and 1.87% (median values) respectively, and ranged from 0 to 6.66%. In addition, the deduced amino acid sequence variability of NC and p6 within mothers (B, C, D, E, F, and H) differed by 0, 0.80, 0.81, 2.47, 0.81, and 4.12% (median values) respectively, ranging from 0 to 13.05%. Furthermore, the infants' (B, C, D, E, F, I1H, and I2H) amino acid sequences varied by 0, 4.05, 1.63, 1.63, 2.45, 0, and 2.04% (median values) respectively, and ranged from 0 to 9.31%. The amino acid sequence variability between epidemiologically linked mother-infant pairs (pairs B, C, D, E, F, and H) varied by 0, 5.74, 1.63, 2.47, 3.28, and 3.28% (median values) respectively, and ranged from 0 to 14.55%. The nucleotide and amino acid sequence variability was also calculated between epidemiologically unlinked individuals. It was determined that the nucleotide distances gave a median value of 7.68, while the amino acid distances produced a median of 14.68. A comparison revealed that the variability between epidemiologically linked mother-infant pairs was lower than the variability between epidemiologically unlinked individuals. This suggests that epidemiologically linked sequences were closer to each other evolutionarily than to unlinked sequences.

**Table 1:** Patient demographics, clinical, and laboratory parameters of six HIV-1 infected mother-infant pairs involved in vertical transmission.

Patient	Age	Sex	CD4+ cells/mm3	Length of infection	Antiviral Drug	Clinical Evaluation
MB	28 yr	F	509	11 mo	None	Asymptomatic
IB	4.75 mo	M	1942	4.75 mo	None	Asymptomatic
MC	23 yr	F	818	1 yr 6 mo	None	Asymptomatic
IC	14 mo	F	*772*	14 mo	ZDV	Symptomatic AIDS
MD	31 yr	F	480	2 yr 6 mo	None	Asymptomatic
ID	28 mo	M	46	28 mo	ddC	Symptomatic AIDS; failed ZDV therapy
ME	26 yr	F	395	2 yr	ZDV	Symptomatic AIDS
IE	34 mo	M	588	34 mo	ZDV	Symptomatic AIDS
MF	23 yr	F	692	2 yr 10 mo	None	Asymptomatic
IF	1 wk	M	2953	1 wk	ZDV	Asymptomatic
MH	33 yr	F	538	5 mo	None	Asymptomatic
IH1	7 mo	F	3157	7 mo	ACTG152	Hepatosplenomegaly, lymphadenopathy
IH2	7 mo	F	2176	7 mo	ACTG152	Hepatosplenomegaly, lymphadenopathy

M: Mother; I: Infant

Length of infection: The closest time of infection that could be documented was the first positive HIV-1 serology date or the first visit of the patient to the AIDS treatment center, where all the HIV-1 positive patients were referred to as soon as an HIV-1 test was positive. As a result, these dates may not reflect the exact dates of infection.

Clinical evaluation for the infants is based on CDC criteria [70]

Mother and infant samples for each pair were collected at the same time

**Table 2:** Nucleotide and amino acid distances of the NC and p6 sequences from mother sets, infant sets, and between mother-infant pairs.

Nucleotide Distances									
Pair	Within Mother			Within Infant			Between Mother and Infant		

	Min	Med	Max	Min	Med	Max	Min	Med	Max
B	0	0.26	0.80	0	0	0.80	0	0	1.10
C	0	0.53	2.12	0	2.26	3.77	0	2.94	5.44
D	0	0.84	2.03	0	0.88	2.57	0	1.13	3.18
E	0	1.13	2.37	0	1.11	2.39	0	1.12	2.68
F	0	0.27	1.62	0	1.78	3.70	0	1.99	4.82
H	0	1.89	6.30	-	-	-	0	1.62	6.28
I1H	-	-	-	0	0	0.27	-	-	-
I2H	-	-	-	0	3.22	5.03	-	-	-

Total	0	0.53	6.30	0	1.16	5.03	0	1.38	6.28

**Amino Acid Distances**									

Pair	Within Mother			Within Infant			Between Mother and Infant		

	Min	Med	Max	Min	Med	Max	Min	Med	Max
B	0	0	4.12	0	0	1.63	0	0	4.12
C	0	0.8	4.89	0	4.05	7.49	0	5.74	14.55
D	0	0.81	4.12	0	1.63	4.12	0	1.63	5.87
E	0	2.47	6.74	0	1.63	4.97	0	2.47	6.74
F	0	0.81	3.28	0	2.45	5.82	0	3.28	8.43
H	0	4.12	13.05	-	-	-	0	3.28	13.05
I1H	-	-	-	0	0	0	-	-	-
I2H	-	-	-	0	2.04	9.31	-	-	-

Total	0	0.81	13.05	0	1.63	9.31	0	2.45	14.55

M: Mother; I: Infant. Min: Minimum; Med: Median; Max: Maximum.

Totals were calculated for all pairs together.

We also evaluated if the low variability of NC sequences seen in our mother-infant pair isolates was due to errors made by Platinum *Pfx *Taq polymerase used in our study. We did not find any errors made by the Taq polymerase when we used a known sequence of HIV-1 NL4-3 for PCR amplification and DNA sequencing of the NC gene.

### Dynamics of HIV-1 NC and p6 gene evolution in mother-infant pairs

Different models of evolution were suggested by Modeltest 3.06 [47] based on maximum likelihood estimates and chi square tests that were performed by the program. The estimates of genetic diversity of the NC and p6 sequences obtained were determined using the Watterson model, which assumes segregated sites, and the Coalesce model, which assumes a constant population size. These estimates of genetic diversity are displayed as theta values, and represent the rate of mutation per site per generation (Table 3). The Watterson model estimated the level of genetic diversity within infected mothers to be 0.014, and within infected infants to be 0.015. Slightly greater estimates were obtained using the Coalesce method, with the genetic diversity between mothers being 0.014, and between infants 0.029. Together these data suggest that both the mother and infant populations evolved slowly and at similar rates. The difference between the estimates of genetic diversity between the mother and infant sequences, using either method, is not statistically significant.

**Table 3:** Estimates of genetic  diversity of the NC and p6 sequences from six HIV-1 infected mother-infant  pairs involved in vertical transmission.

Patient	Within Mothers		Within Infants	
	θ_W_	θc	θ_W_	θc
B	0.004	0.009	0.003	0.002
C	0.011	0.017	0.031	0.075
D	0.022	0.017	0.011	0.020
E	0.016	0.015	0.015	0.024
F	0.009	0.011	0.025	0.061
H	0.023	0.016	-	-
I1H	-	-	0.001	0.001
I2H	-	-	0.017	0.017

Total	0.014	0.014	0.015	0.029

θ_W_: viral diversity as estimated by the Watterson method.

θ_C_: viral diversity as estimated by the Coalesce method.

Totals were calculated as the average of all values.

### Rates of accumulation of non-synonymous and synonymous substitutions

The ratio of the accumulation of non-synonymous (dn) to synonymous substitutions (ds) was used to estimate the selection pressure on the NC and p6 gene by using a model modified by Nielson and Yang [48], which was then implemented by codeML [49]. The advantage of the codeML method lies in the fact that this model views the codon as the unit of evolution, as opposed to the nucleotide which is used in other models [50]. Moreover, the Nielson and Yang model does not assume that all sites within a sequence are under the same selection pressure. This gives a more realistic view of evolution because mutations, in some cases leading to only a single amino acid change, can be more advantageous or deleterious in some regions of a protein compared to others, and thus undergoes positive or purifying selection. In addition the dn/ds ratio that is calculated determines the selection pressure acting upon the changes within the codon, with a dn/ds ratio of greater than 1 indicating that positive selection pressure is present. Not only does this model determine positive selection pressure, it also calculates the percentage of mutations that are selected. The percent of mutations that are conserved fall in the p1 category, the neutral mutations are in the p2 category, and the positively selected mutations are in the p3 category. The estimations of the dn/ds ratio as well as the percentages in each category (p1, p2, and p3) for each patient sample are given in Table 4. All of the sequence populations analyzed displayed a dn/ds ratio greater than or equal to 1.

In general, the mother sequences displayed a higher percentage of positively selected p3 sites compared to the infants. Within mothers, almost 100% of the mutations in mothers B, C, and F were positively selected. Although mother D and mother H have the highest dn/ds values, less than 1% of the mutations are positively selected. Most of the mutations in mother D and mother H are neutral. When compared to the mothers, infants have less than 3% of mutations that are positively selected, with the exceptions of infant D and the second infant H twin (I2H). In contrast to the mothers, the infants have a more even distribution of conserved and neutral mutations. It is interesting to note that in four of the seven infants, over 50% of the mutations observed were neutral mutations. This higher proportion of p2 sites in infants was also seen in analysis of the *nef *and reverse transcriptase (RT) genes [12,51]. The positive selection pressure acting on these patient sequences was estimated in codeML using both neutral models and positive selection models. In patients where a substantial proportion of mutations were in the p3 category, the positive selection model was significant over the neutral model (data not shown). These data indicate that a higher percentage of mutations are positively selected in mothers as compared to infants, however positive selection pressure was observed when analyzing the NC gene sequences from both the mother and infant patient samples.

### Analysis of functional domains of NC and p6 within mother-infant pairs

The function of the HIV-1 NC protein is to bind to viral RNA and DNA. This protein contains two zinc fingers and many basic amino acids that allow it to interact with the viral nucleic acids. The critical residues of the zinc fingers consist of three cysteines and one histidine, and have the sequence C-X_2_-C-X_4_-H-X_4_-C, with X representing any amino acid, and are located at positions 16 to 29 and 37 to 50 within the NC protein [20]. The critical residues within these zinc fingers are located at positions 16, 19, 24, and 29 in the first zinc finger and positions 37, 40, 45, and 50 in the second zinc finger. A mutation at any of these critical residues abolishes the ability of these functional domains to bind the zinc cofactor, which will lead to improper folding of the protein [24,29]. Analysis of the first zinc finger sequence from the six mother-infant pairs shows that of the 168 sequences acquired, only two contained mutations at the critical residues (Figs. 2, 3, 4). Infant C clone 2 (IC-2) contained the substitution C19R, and mother B clone 2 (MB-2) (Fig. 2) contained the substitution H24Y. Furthermore, the second zinc finger contained substitutions at the critical residues in only one clone; infant C clone 3 (IC-3) contained an H45Y substitution (Fig. 2). However some sequences within mother H and the second infant H twin (I2H) contain substitutions that resulted in the formation of a stop codon at position 38 within the second zinc finger (Fig. 4). These stop codons would result in a truncation in the second zinc finger, and would result in only one functional zinc finger (the first zinc finger) within the NC protein of these clones. When two zinc fingers are present, the first generally tends to play a more critical role [18,20], however removal of the second zinc finger function has been shown to greatly decrease the annealing capacity of the NC protein [20,29]. Despite these exceptions, the critical residues of both zinc fingers within the mother-infant NC sequences were highly conserved.

There are several basic residues, arginine (R), lysine (K), or histidine (H), within the NC protein that also allow it to function. Of the 56 amino acids that make up the NC protein, 17 are basic [21]. These basic residues spread throughout the protein and are responsible for interacting with the side chains on viral nucleic acids [18,52]. Mutations in these basic residues has been shown to reduce RNA binding and encapsidation [21]. Analysis of the sequences from the mother-infant pairs shows that there are substitutions at many of the basic residues. However looking more in depth, a majority of the substitutions are from one basic amino acid to another. Furthermore, there are several substitutions from non-basic to basic residues throughout the protein sequences obtained, and some of these substitutions are compensatory mutations for changes from a basic amino acid elsewhere within the sequence (Figs. 2, 3, 4). While there are several substitutions involving basic amino acids within the NC protein sequences from the six mother-infant pairs, the presence of several basic residues throughout the protein sequences is highly conserved.

The p6 gene was also sequenced as a result of sequencing the NC gene. The p6 protein contains two major functional domains, the viral late domain located at positions 79 to 83, and the Vpr binding domains located at positions 87 to 90 and 107 to 118 [30,33,45]. The late domain contains the sequence proline-threonine-alanine-proline-proline (PTAPP) and is responsible for ensuring proper budding of a newly formed virion from the host cell membrane [32,53]. The prolines at positions 82 and 83 have especially been shown to be critical for Tsg101 binding [32]. Analysis of the p6 protein sequences from the six mother-infant pairs revealed that the late domains, especially the critical prolines, are conserved in most of the sequences obtained (Figs. 2, 3, 4). Interestingly, in several sequences from mother D there is a duplication of the late domain (Fig. 3). It has been shown that duplication of this domain could be linked to antiretroviral drug resistance [54,55]. However since mother D has not been exposed to antiretroviral drugs (Table 1), this duplication must have arisen naturally or was present in the virus that was initially transmitted to mother D. In general, the late domain of the p6 protein from the mother-infant pairs was highly conserved.

The Vpr binding domain could be located in two possible positions within the p6 protein sequences of the six mother-infant pairs, either positions 87 to 90 or 107 to 118 [30,33,45] (Fig. 2). The domain located at positions 87 to 107 has the sequence phenylalanine-arginine-phenylalanine-glycine (FRFG) [30], while the domain at positions 107 to 118 has the sequence leucine-XX-leucine-XX-leucine-XX-leucine-XX ((LXX)_4_)[45], with X representing any amino acid. These Vpr binding domains are responsible for inclusion of the viral accessory protein Vpr into newly forming virions. Analysis of the protein sequences from the mother-infant pairs revealed that while the FRFG Vpr binding domain was mostly conserved, there were some notable exceptions. There were single amino acid substitutions within the domain in every clone of mother and infant F (pair F), infant C (IC), and infant D (ID) (Figs. 2, 3, 4). It has been shown that mutations at either of the two phenylalanines within the FRFG domain, which is seen in pair F and infant D, causes a loss of Vpr packaging within virions; while a substitution at the arginine site, which is seen in infant C, seems to have little to no effect [30]. In spite of these exceptions however, the FRFG Vpr binding domain within the six mother-infant pairs analyzed was mostly conserved. Analyzing the protein sequences also showed that the (LXX)_4 _domain was also mostly conserved within the sequences obtained, except for the first leucine in every clone. This first leucine was substituted with either a methionine (M), a valine (V), a histidine (H), an arginine (R), or a glutamine (Q) (Figs. 2, 3, 4). A change in this first leucine has been shown to decrease Vpr binding [45]. The third and fourth leucine have been shown to be critical for Vpr inclusion [33,34], and these residues are highly conserved within the mother-infant sequences obtained. As with the FRFG domain, the (LXX)_4 _Vpr binding was mostly conserved within the sequences of the mother-infant pairs analyzed.

The p6 gene product also contains a region, from amino acid positions 31–46 with the sequence DKELYPLASLRSLFG that is responsible for interacting with the host cell factor AIP1 [31]. This motif within the mother-infant pair sequences was mostly conserved, however every clone analyzed contained a substitution at the first leucine, as also seen in the (LXX)_4 _domain (Figs 2, 3, 4). Mother and infant C (pair C) (Fig. 2) and mother and infant D (pair D) (Fig. 3) also contained additional substitutions within the AIP1 binding domain. It is not known at this time what effect these substitutions would have on the interaction of p6 with AIP1. Despite these exceptions, the AIP1 binding domain was mostly conserved within the six mother-infant pairs' sequences obtained.

In addition to the substitutions mentioned, there were several other substitutions that occurred outside of the functional domains. The effect that these changes would have is not known at this time.

### Analysis of immunologically relevant mutations within the CTL epitopes of NC and p6

The cytotoxic T-lymphocyte (CTL) response is known to contribute a significant portion of the body's immune response to an HIV-1 infection. In patients with a strong CTL response, there has been shown to be a decrease in the viral load within the patient and a long-term non-progressor disease status [56]. It has also been shown that mothers who transmit the virus to their infants have an increased number of CTL escape variants when compared to non-transmitting mothers [57]. This could demonstrate a correlation between the amount of CTL escape variants circulating in the mothers' bloodstream and the likelihood of vertical transmission of the virus. There have been several CTL epitopes identified within the NC and p6 proteins. The first epitope within the NC protein has the sequence CRAPRKKGC and is located between amino acid positions 28 and 36 [58]. This epitope is recognized by HLA-B14 and contains the last cystine of the first zinc finger, and the first cystine of the second zinc finger. Analysis of the NC amino acid sequences from the six mother-infant pairs revealed that this epitope was highly conserved in most of the clones that were obtained (Figs. 2, 3, 4). Another CTL epitope, KEGHQMKDCTERQANF, is located at amino acid positions 42–57 and is recognized by several HLA types [58]. This epitope spans the last 14 amino acids of the NC protein and contains the histidine and final cystine of the second zinc finger. Again this epitope was mostly conserved when the sequences from the mother-infant pairs was analyzed. The next motif, CTERQANFL, is located from positions 50 to 56 and is recognized by HLA-B61 [58]. This epitope contains the last cystine of the second zinc finger and was highly conserved within the mother-infant sequences obtained.

The first motif within the p6 gene sequences, GNFLQSRPEPTAPPF, is located at amino acid positions 70–84 and is recognized by several HLA types [58]. Analysis of the mother-infant sequences revealed that this epitope was mostly conserved, with the exception of mother and infant C (pair C). Pair C contains a PTV insertion beginning at position 78 (Fig. 2). It is not known at this time what effect on CTL recognition this insertion would have. The next epitope is located at amino acid positions 105 to 114 and has the sequence KELYPLTSL [58]. This epitope is recognized by HLA-B60 and is positioned within the (LXX)_4 _Vpr binding domain. Within the mother-infant sequences obtained this CTL epitope was mostly conserved, however the first lysine within the epitope was substituted within every clone analyzed (Figs. 2, 3, 4). It is not known at this time what effect this substitution would have on recognition of this epitope. Another epitope, YPLTSLRSLF, is located at positions 108 to 117 and is recognized by HLA-B7 [58]. Analysis of the six mother-infant pairs' sequences revealed that this epitope was mostly conserved (Figs. 2, 3, 4). Overall, analysis of the CTL recognition epitopes within the sequences of the mother-infant pairs' displayed that these epitopes which are involved in immune recognition of the virus were mostly conserved.

## Discussion

In this study, we have shown that the *gag *p17, NC and p6 genes, of HIV-1 were mostly conserved during vertical transmission. Six mother-infant pairs were analyzed and the NC and p6 open reading frames were found to be conserved with a frequency of 92.8%. When distance analysis and population dynamics were performed, it was found that there was a low degree of viral heterogeneity as well as genetic diversity. In spite of this, a positive selection pressure was found be to acting on the NC and p6 gene sequences. Comparison of the mother-infant pairs to each other revealed that the NC and p6 sequences from epidemiologically linked individuals were more closely related to each other than to epidemiologically unlinked individuals. While the individual pairs could clearly be distinguished from one another based on sequence analysis, the functional domains within all sequences analyzed remained mostly conserved. These findings suggest that maintenance of conserved NC and p6 genes during vertical transmission is important for pathogenesis of HIV-1 in mothers and infants.

The open reading frames (ORF) of the NC and p6 genes sequenced from six mother-infant pairs involved in vertical transmission were highly conserved. Of the 168 sequences analyzed, 156 contained an intact ORF (Fig 2). The remaining sequences contained a substitution that resulted in the formation of a premature stop codon. This data is comparable to previously analyzed genes, including *gag *p17, *vif, vpr, tat, vpu, nef*, and reverse transcriptase [12-16,51,59,60]. Maintenance of the NC and p6 ORFs confirms the importance of this gene in the viral lifecycle, and suggests its importance in vertical transmission of the virus.

Several pair and patient specific sequence motifs were observed when a multiple sequence alignment was performed. There were also three substitutions that were present in every sequence analyzed (Figs 2, 3, 4). These universal mutations could provide a possible target for future diagnosis or for antiretroviral therapy development. The NC has already become a promising target for antiviral therapy [24,61-63], perhaps the information that this study provides can help further the knowledge of HIV-1 vertical transmission and help to design preventative methods. Analysis of the nucleotide and amino acid distances revealed a low degree of viral heterogeneity within the sequences analyzed (Fig 3). A low degree of genetic diversity was also found by the Watterson and Coalesce methods (Fig 4). The mutation rate per site per generation (the θ value) was slightly, although not significantly, higher in infants than in mothers. This slight increase in mutation rate could account for the slightly higher heterogeneity within the infant sequences. These values could be higher within the infants due to the fact that the virus within the infants has had less time to adapt to its new host than in the mothers. This is also supported by the observation that mothers have a higher percentage of mutations that are positively selected as compared to infants, where most of the mutations were neutral or conserved. A higher percentage of positively selected mutations could indicate that the virus has evolved for a longer duration in the mothers. Since it is likely that the infants have been infected for a shorter duration than the mothers, the viral variants in the infants have been exposed to the selection pressure of the new host for a shorter time. Although the distance values indicate that the infants were slightly more diverse, phylogenetic analysis reveals that each of the six mother-infant pairs clustered together in distinct subtrees (Fig 1). This reveals that although the virus is evolving in separate hosts, it is still more similar to the virus in its epidemiologically linked host, than to the virus in an epidemiologically unlinked host. Information of this kind can be helpful in establishing an epidemiological relationship between transmitter-recipient pairs.

The functional domains in the NC and p6 genes from the six mother-infant pairs were mostly conserved during vertical transmission. The critical residues of the NC zinc fingers were highly conserved, while the basic residues throughout the NC protein displayed more variability. However, these changes within the basic residues did not result in an overall loss of these basic amino acids. In fact, most of the basic amino acids that were substituted were replaced by another basic residue. While some changes did result in a loss of a basic residue, other compensatory mutations elsewhere in the amino acid sequence replaced the residue lost. This conservation of critical zinc finger residues and presence of many basic residues implies that it is very important to the virus evolutionarily to maintain the function these motifs provide. The viral late domain, Vpr binding sites, and AIP1 binding site were all mostly conserved as well, when the mother-infant p6 sequences were analyzed. Critical residues within the late domain, including the two prolines vital for Tsg101 binding, were highly conserved during vertical transmission. This domain was not only conserved, but duplicated within some mother D sequences. It has been shown that duplication of this domain could be linked to antiretroviral drug resistance [54,55,64]. However since mother D has not been exposed to antiretroviral drugs (Table 1), this duplication must have arisen naturally or was present in the virus that was initially transmitted to mother D. It is not known at this point, however, what effect this duplication would have on the budding ability of the virus. Both of the Vpr binding domains were mostly conserved in the mother-infant sequences obtained, as was the AIP1 binding motif. Again, the conservation of these functional domains during vertical transmission suggests that it is important to the virus evolutionarily to maintain the functions that these regions provide.

Several substitutions were seen outside of the functional domains mentioned. While the relevance of these changes is not known at this time, the effects could be studied by performing biological studies using the NC clones obtained within this study. Another study of interest would be to characterize the same NC region in mothers who naturally failed to transmit the virus to their offspring and compare the results to that of this study.

There were several CTL epitopes, which were recognized by several different HLA types, within the mother-infant NC sequences obtained. It has been shown that transmitting mothers have larger numbers of CTL escape variants as compared to non-transmitting mothers, but the transmitted viruses carrying epitopes are not escape variants [57]. It is entirely possible however, that the CTL responses studied are tissue specific. A representation of peripheral blood, and the virus and CTL variants in the placenta, birth canal, and breast milk are different [65]. There were substitutions within the CTL epitopes of the NC sequences from the six mother-infant pairs; however it is unknown what effect these changes may have. It is possible that the substitutions observed in this study may influence vertical transmission by resulting in differential responses in a tissue specific manner.

Although great strides have been made in the prevention of HIV-1 vertical transmission in countries such as the United States, the infection of children vertically in developing countries remains a large problem. In order deal with this problem, a better understanding of the mechanisms involved needs to be established. A characterization of many of the viral genes during vertical transmission has already been completed [1,2,11,12,14-16,51,59,60], and has shed new light on the molecular mechanisms of an HIV-1 vertically acquired infection. Using this previous information, new targets and strategies may be able to be developed in order to prevent the spread of this disease. The data presented in this study may be able to provide new insights into the molecular characteristics of HIV-1 vertical transmission and further our understanding so that novel treatment and prevention strategies may be developed.

## Conclusion

In this study we have shown that the HIV-1 NC and p6 genes were mostly conserved in six mother-infant pairs following vertical transmission. Phylogenetic analysis revealed that virus from epidemiologically linked mother-infant pairs was closer to each other than to epidemiologically unlinked pairs. The NC and p6 open reading frames were highly conserved and estimates of viral heterogeneity and genetic diversity were low. Several patient and pair specific substitutions were seen within the NC amino acid sequence. The functional domains within the NC and p6 sequences, which include two zinc fingers, several basic residues, a viral late domain, two possible Vpr binding sites, and a binding site for AIP1, were widely conserved following vertical transmission. The data presented in this study provides evidence that supports the critical role of the NC gene product in the viral lifecycle and in pathogenesis of HIV-1 during vertical transmission.

## Materials and methods

### Patient population

Blood samples were collected from six mother-infant pairs following vertical transmission, including a set of twins (I1H and I2H) in the case of mother H. The demographics, clinical and laboratory findings on these mother-infant pairs is summarized in Table 1. The Human Subjects Committee of the University of Arizona (Tucson, AZ) and the Institutional Review Board of the Children's Hospital Medical Center (Cincinnati, Ohio) approved this study. Written informed consent to participate in this study was obtained from the mothers of the mother-infant pairs.

### PCR amplification, cloning, and sequencing

Peripheral blood mononuclear cells (PBMCs) were isolated using a single-step Ficoll-Hypaque method (Pharmacia-LKB) from whole blood samples of HIV-1 infected mother-and-infant pairs that were involved in vertical transmission. The PBMCs DNA, which contains the integrated HIV-1 genome, was isolated as previously described [9]. The HIV-1 NC gene was amplified using a two-step polymerase chain reaction (PCR) method according to the modified protocol described by Ahmad et al [9]. Outer primers NC-1 (5'GAAGAAATGATGACAGCATGTCAGGGAGTGGG, 1819 to 1851, sense) and NC-2 (5'CCATCTTCCTGGCAAATTCATTTCTTCTAATACT, 2344 to 2378, antisense) were first used, followed by nested primers NC-3 (5'CACCGGCCATAAAGCAAGAGTTTTGGCTGAAGC, 1854 to 1887, sense) and NC-4 (5' CATCTGCTCCTGTATCTAATAGAGCTTCCTT, 2310 to 2341, antisense). Equal amounts of PBMC DNA (approximately 25 to 50 copies, minimum) as determined by end-point dilution was subjected to multiple (four to six) PCRs to obtain clones that were sequenced and analyzed. PCR was carried out in a 25μl reaction mixture containing 2.5 μl 10× *Pfx *Amplification buffer, 2.5 mM MgSO_4_, 400 μM of each dATP, dCTP, dGTP, and dTTP, 0.2 μM of each outer primer, and 2.5 units (U) of Platinum *Pfx *DNA polymerase (Invitrogen Inc.). The reaction was initiated at 94°C for 2 minutes (min), and then cycled at 94°C for 30 seconds (sec), 50°C for 30 sec, and 72°C for 2 min, for 35 cycles, with an addition extension period of 10 min at 72°C to end the reaction. Following the first round of PCR, 5–8 μl of the first-PCR product was used for nested PCR using the same reagents and the inner primers. This nested PCR was cycled at 94°C for 30 sec, 55°C for 30 sec, and 72°C for 2 min, for 35 cycles. A negative control was included with each PCR which used sterile water in place of DNA. PCR was also performed on HIV-1 NL4-3, of which the sequence is known (GenBank accession number M19921), to assess any errors made by the Platinum *Pfx *DNA polymerase. To avoid contamination, all samples, reagents and PCR products were kept separately and dispensed in a separate room free of all laboratory-used DNA.

The PCR products were visualized on a 1% agarose gel and cloned into the TOPO TA cloning system (pCR 2.1-TOPO vector) as per manufacturer's instructions (Invitrogen Inc.). Positive bacterial colonies were determined by blue-white screening, and the presence of correct-sized inserts was confirmed by restriction digest. Eight to eighteen clones from each mother and infant, from multiple independent PCRs, were sequenced using the Thermosequenase Cycle Sequencing protocol (USB) and the University of Arizona Biotechnology Center automated system (ABI Prism^® ^3700 DNA automated sequencing system).

### Sequence analysis

The nucleotide sequences of the HIV-1 NC gene (approximately 375 bp) from six mother-infant pairs were analyzed using the Wisconsin package version 10.1 of the Genetics Computer Group (GCG) and were translated to corresponding deduced amino acid sequences (125 amino acids). Both the nucleotide and amino acid sequences were aligned, using the HIV-1 NL4-3 NC sequence as a reference, by Clustal X. A model of evolution was optimized for the entire nucleotide sequence data set using the Huelsenbeck and Crandall approach [66]. Likelihood models of evolution were calculated using PAUP [46] and a chi square (χ^2^) test was performed using Modeltest 3.06 [47]. The model with the highest likelihood was incorporated into PAUP to generate a neighbor-joining tree, which was bootstrapped 1000 times to ensure fidelity. The tree was based on the nucleotide sequences from the six mother-infant pairs, as well as HIV-1 NL4-3 which was used as a reference sequence and the outgroup for the tree. Using Modeltest and the Akaike Information Criterion (AIC) [67], all the null hypotheses were rejected except the best-fit model (GTR+G). The base frequencies for the NC gene were calculated as: freq A = 0.37, freq C = 0.23, freq G = 0.21 and freq T = 0.19. Five rate categories were also figured, and were as follows: R(A- C)= 1.00, R (A-G)= 5.34, R(A-T) = 0.79, R(C-G) = 0.79, R(C-T) = 5.34, R(G-T) = 1.00. The rate heterogeneity was taken into account using a gamma distribution with a shape parameter (α) of the distribution estimated from the data via maximum likelihood. This shape parameter had a value of 0.7085. A model of evolution for each patient was also generated and optimized to estimate corrected pairwise nucleotide distances using PAUP [46]. Amino acid distances were also estimated using the Jukes-Cantor model within the Wisconsin package version 10.1 of GCG. The minimum, median, and maximum distance values were calculated for both nucleotides and amino acids for each patient as well as for the linked and unlinked patient pairs. The dynamics of HIV-1 evolution was assessed using techniques of population genetics. In population genetics, genetic diversity is defined as θ = 2N_e_μ, where N_e _is the effective population size and μ is the per nucleotide mutation rate per generation. The Watterson model, which assumes segregated sites, and the Metropolis-Hastings model, which assumes a constant population size, was used to estimate the differences in genetic diversity using the Coalesce 1.5 program [68,69]. To analyze the evolutionary processes acting upon the NC gene, we estimated the ratio of nonsynonymous (dn) to synonymous (ds) substitutions by a maximum likelihood model using codeML, which is part of the PAML package [49]. The Nielsen and Yang [48] model considers the codon instead of the nucleotide as the unit of evolution and thus incorporates three distinct categories of sites. The first category represents the sites that are invariable or conserved (p1, dn/ds = 0); the second category represents sites that are neutral (p2, dn/ds = l), at which dn and ds are fixed at the same rate; and the third category represents sites that are under positive selection, where dn has a higher fixation rate than ds (p3, dn/ds >1). The dn/ds was estimated for each patient using both neutral and positive selection models in codeML.

### Nucleotide sequence accession numbers

The nucleotide accession numbers of the sequences submitted to GenBank are DQ026833 through DQ027000.

## Competing interests

The author(s) declare that they have no competing interests.

## Authors' contributions

BW carried out the PCR, cloning, and sequencing. BW, VS, and RR performed the sequence analysis by computer programs. BW and NA participated in the experimental design, data interpretation, and writing of the manuscript. All authors read and approved the final manuscript.

**Table 4:** Ratio of nonsynonymous (dn) to synonymous (ds) substitutions in NC and p6 sequences from six HIV-1 infected mother-infant pairs involved in vertical transmission.

Pair	Mothers					Infants				
	N	p1	p2	p3	dn/ds	N	p1	p2	p3	dn/ds

B	13	0	0	100	1.37	11	0	100	0	1.00
C	9	0	0	100	1.51	15	11.72	86.09	2.19	22.84
D	17	0	99.02	0.98	68.79	12	87.26	0	12.74	6.75
E	11	83.68	0	16.32	33.75	12	48.04	51.02	0.94	37.57
F	18	0.03	0	99.97	1.20	15	21.48	77.20	1.32	9.00
H	16	0	100	0	89.00	-	-	-	-	-
I1H	-	-	-	-	-	11	100	0	0	21.24
I2H	-	-	-	-	-	8	68.65	0	31.34	5.47

Total	84	13.95	33.17	52.88	32.60	84	48.16	44.90	6.94	14.82

N: Number of clones sequenced, Totals were calculated as the average of all values.

p1: proportion of conserved codons as a percent

p2: proportion of neutral codons as a percent

p3: proportion of positively selected codons as a percent; dn/ds = dn/ds ratio at p3
